# Systemic factors related to soluble (pro)renin receptor in plasma of patients with proliferative diabetic retinopathy

**DOI:** 10.1371/journal.pone.0189696

**Published:** 2017-12-14

**Authors:** Keitaro Hase, Atsuhiro Kanda, Ikuyo Hirose, Kousuke Noda, Susumu Ishida

**Affiliations:** Laboratory of Ocular Cell Biology and Visual Science, Department of Ophthalmology, Faculty of Medicine and Graduate School of Medicine, Hokkaido University, Sapporo, Hokkaido, Japan; Monash University, Melbourne, AUSTRALIA

## Abstract

(Pro)renin receptor [(P)RR], a new component of the tissue renin-angiotensin system (RAS), plays a crucial role in inflammation and angiogenesis in the eye, thus contributing to the development of proliferative diabetic retinopathy (PDR). In this study, we investigated systemic factors related to plasma levels of soluble form of (P)RR [s(P)RR] in patients with PDR. Twenty type II diabetic patients with PDR and 20 age-matched, non-diabetic patients with idiopathic macular diseases were enrolled, and plasma levels of various molecules were measured by enzyme-linked immunosorbent assays. Human retinal microvascular endothelial cells were stimulated with several diabetes-related conditions to evaluate changes in gene expression using real-time quantitative PCR. Of various systemic parameters examined, the PDR patients had significantly higher blood sugar and serum creatinine levels than non-diabetic controls. Protein levels of s(P)RR, prorenin, tumor necrosis factor (TNF)-α, complement factor D (CFD), and leucine-rich α-2-glycoprotein 1 (LRG1) significantly increased in the plasma of PDR subjects as compared to non-diabetes, with positive correlations detected between s(P)RR and these inflammatory molecules but not prorenin. Estimated glomerular filtration rate and serum creatinine were also correlated with plasma s(P)RR, but not prorenin, levels. Among the inflammatory molecules correlated with s(P)RR in the plasma, TNF-α, but not CFD or LRG1, application to retinal endothelial cells upregulated the mRNA expression of (P)RR but not prorenin, while stimulation with high glucose enhanced both (P)RR and prorenin expression. These findings suggested close relationships between plasma s(P)RR and diabetes-induced factors including chronic inflammation, renal dysfunction, and hyperglycemia in patients with PDR.

## Introduction

Diabetes mellitus (DM) affects over 415 million people worldwide [1], approximately one-third of whom will eventually develop diabetic retinopathy (DR), one of the most common complications in diabetes. DR, a form of microangiopathy in the retina, is one of the leading causes of severe vision loss and blindness when it progresses to the stage of proliferative DR (PDR) characterized by aberrant retinal neovessel growth, *i*.*e*., fibrovascular proliferation. Increasing evidence has suggested that chronic inflammation with leukocyte infiltration plays a pivotal role in the pathogenesis of DR [2, 3], which is now regarded as an inflammatory as well as angiogenic disorder. Inflammatory leakage from dilated hyperpermeable vasculature causes the entry of lipid- and protein-containing fluid into the retinal parenchyma, thus generating commonly seen DR lesions such as hard exudates and macular edema.

The tissue renin-angiotensin system (RAS), unlike the circulatory RAS for regulating systemic blood pressure, causes tissue-specific malfunctions involved in inflammation, angiogenesis and fibrosis in various organs including the eye [4–6]. Several clinical trials showed that inhibiting the RAS using an angiotensin II type 1 receptor blocker successfully suppressed the incidence and progression of DR [7, 8]. (Pro)renin receptor [(P)RR] binds with prorenin to convert it into non-proteolytically activated prorenin that exerts renin enzymatic activity, thereby triggering the tissue RAS [9]. (P)RR also exists in its soluble form called s(P)RR in the systemic circulation after cleavage by proteases such as furin [10]. Interestingly, prorenin is capable of binding with s(P)RR as well as membrane-bound (P)RR, both of which initiate the RAS cascade via the non-proteolytic conversion of prorenin into activated prorenin [11]. We reported that the elevated levels of s(P)RR in PDR eyes, released from neovascular endothelial cells in fibrovascular tissues, showed a positive correlation with the angiogenic activity of PDR [4, 12]. Previous reports demonstrated that increased s(P)RR and prorenin levels in the plasma or serum were associated with various diseases including obesity, hypertension, preeclampsia, and heart and kidney failure [13–20]. To the best of our knowledge, however, no data have shown s(P)RR concentration in the systemic circulation of patients with PDR.

In this study, we investigated whether plasma levels of s(P)RR, prorenin, and activated prorenin increase in PDR patients, and explored cause-effect relationships between these RAS initiators and diabetes-induced systemic factors such as chronic inflammation.

## Materials and methods

### Human blood samples

Peripheral blood samples were collected preoperatively from 20 type 2 diabetic patients with PDR (13 females and 7 males, PDR group) and 20 non-diabetic, age-matched patients with idiopathic macular diseases including epiretinal membrane and macular hole (10 females and 10 males, non-DM group) [21]. To obtain plasma, we centrifuged the blood samples at 3,000 rpm for 10 min at 4°C. The plasma was then carefully transferred into polypropylene tubes and stored at -80°C. To purify RNAs, blood samples were collected in PAXgene blood RNA tubes (BD Biosciences, San Jose, CA, USA) following the manufacturer’s protocol. Systemic and ophthalmic characteristics of the subjects enrolled in this study are shown in Table 1. Ocular parameters listed here represent preoperative data obtained from unilateral eyes having received vitrectomy surgery. This study was conducted in accordance with the tenets of the Declaration of Helsinki. After receiving approval from the institutional review board of Hokkaido University Hospital, written informed consent was obtained from all patients (IRB No. 011–0172).

**Table 1:** Basal characteristics of participating patients.

Patients	Non-DM	PDR	*p* value
**Number (persons)**	20	20	-
**Age (years)**	63.35 ± 0.96	62.40 ± 1.95	0.665
**Gender (% male)**	50	35	0.523^#^
**RBS (mg/dl)**	105.90 ± 2.95	159.40 ± 12.02	< 0.001
**HbA1c (%)**	Not tested	7.12 ± 0.28	-
**Duration (years)**	Not applicable	12.94 ± 1.89	-
**HT (%)**	45	55	0.752^#^
**BMI**	23.60 ± 0.73	24.28 ± 0.96	0.580
**ARB use (%)**	35	45	0.748^#^
**SBP (mmHg)**	128.50 ± 3.82	132.00 ± 4.49	0.556
**DBP (mmHg)**	71.70 ± 2.04	73.35 ± 3.20	0.666
**sCr (mg/dl)**	0.71 ± 0.03	1.00 ± 0.11	0.024
**eGFR (ml/min/1.73m^2^)**	76.45 ± 3.49	64.02 ± 7.34	0.138
**Log MAR**	0.71 ± 0.09	1.28 ± 0.16	0.003
**IOP (mmHg)**	14.75 ± 0.79	15.00 ± 0.83	0.829
**CRT (μm)**	222.30 ± 63.38	349.80 ± 42.20	0.103

RBS, random blood sugar; Duration, duration of diabetes mellitus; HT, hypertension; BMI, body mass index; ARB, angiotensin II receptor blocker; SBP, systolic blood pressure; DBP, diastolic blood pressure; sCr, serum creatinine; eGFR, estimated glomerular filtration rate; MAR, minimum angle of resolution; IOP, intraocular pressure; CRT, central retinal thickness. Results are expressed as mean ± SEM. n = 20 in each group. Statistical analyses were performed using Student’s t test or ^#^Fisher’s exact test.

### Enzyme-linked immunosorbent assays (ELISA)

The protein levels of prorenin, s(P)RR and leucine-rich α-2-glycoprotein 1 (LRG1) in the plasma were determined with human prorenin (Abcam, Cambridge, UK), s(P)RR and LRG1 (Immuno-Biological Laboratories, Gunma, Japan) ELISA kits per the manufacturers’ instructions. The optical density was determined using a micro-plate reader (Sunrise, TECAN, Männedorf, Switzerland). The levels of proteins [tumor necrosis factor (TNF)-α, complement factor D (CFD), vascular adhesion protein (VAP)-1, monocyte chemoattractant protein-1/C-C motif ligand 2 (MCP-1/CCL2), interferon (IFN)-γ, interleukin (IL)-6, vascular cell adhesion molecule (VCAM)-1, intercellular adhesion molecule (ICAM)-1, fibroblast growth factor (FGF)-1, FGF-2, placental growth factor (PlGF), endostatin, and vascular endothelial growth factor (VEGF)] were determined by magnetic multiplex bead-based quantitative immunoassay using the MAGPIX (Millipore, Austin, TX, USA) and Luminex assay kit (R&D Systems, Minneapolis, MN, USA), according to the manufacturers’ protocol.

Activated prorenin corresponds to prorenin bound with s(P)RR, and the dissociation constant (K_D_) for the binding of prorenin with s(P)RR was calculated in a previous report [22] as follows: K_D_ (4.0 nmol/l) = [prorenin] x [s(P)RR] / [activated prorenin]. Based on the K_D_, we determined the plasma concentration of activated prorenin.

### Cell culture and chemicals

Human retinal microvascular endothelial cells (HRMECs; Cell Systems Corporation, Kirkland, WA, USA) were cultured in CS-C complete medium (Cell Systems Corporation) containing 5-mM glucose, which corresponds to blood glucose concentration under normal conditions. For high-glucose stimulation, HRMECs were cultured in CS-C complete medium containing 30-mM glucose, which corresponds to a blood glucose concentration under hyperglycemia.

After serum starvation, HRMECs were treated with recombinant human TNF-α (PeproTech, Rocky Hill, NJ, USA), CFD (Thermo Fisher Scientific, Waltham, MA, USA), and LRG1 (R&D Systems) for 24 h and processed for analysis to detect mRNA expression levels. For the TNF-α neutralization bioassay, recombinant human TNF-α at 20 ng/ml was pre-incubated with rabbit anti-TNF-α neutralizing antibody (Cell Signaling Technology, Danvers, MA, USA) for 15 min at 37°C. After pre-incubation, the cells were treated for 24 h and processed for analysis to detect gene expression levels. Normal rabbit IgG (R&D Systems) was used as the control for the anti-TNF-α neutralizing antibody.

To cover the handle region of the prorenin molecule, which is the binding site of (P)RR [4], we synthesized decoy peptides NH_2_-RIFLKRMPSI-COOH as a human (P)RR blocker (PRRB), and purified them by high-pressure liquid chromatography on a C-18 reverse-phase column. After the cells were serum-deprived, HRMECs were pretreated with 1-μM PRRB or 10-μM losartan (Sigma-Aldrich, St. Louis, MO, USA) as an angiotensin II type 1 receptor blocker for 1 h. Prorenin or angiotensin II was then added at the final concentration of 10 nM or 1 μM, respectively. Cells were incubated for 24 h and processed for analysis to detect RNA expression levels.

### Real-time quantitative PCR (qPCR)

Total RNA isolation and reverse transcription were performed from cells using SuperPrep Cell Lysis & RT Kit for qPCR (TOYOBO, Tokyo, Japan) and from peripheral whole bloods using PAXgene Blood RNA Isolation Kit (QIAGEN, Venlo, The Netherlands) and GoScrip Reverse Transcriptase (Promega, Madison, WI) following the manufacturers’ protocols. The following primers for genes were used: *ATP6AP2* [(P)RR; forward 5′-AGG CAG TGT CAT TTC GTA CC-3′, reverse 5′-GCC TTC CCT ACC ATA TAC ACT C-3′], *TNFA* (TNF-α; forward 5′-ACT TTG GAG TGA TCG GCC-3′, reverse 5′-GCT TGA GGG TTT GCT ACA AC-3′), *CFD* (forward 5’-GAC ACC ATC GAC CAC GAC-3′, reverse 5′-GTT GAC TAT GCC CCA GCC-3′), *LRG1* (forward 5′-GTT GGA GAC CTT GCC ACC T-3′, reverse 5′-GCT TGT TGC CGT TCA GGA-3′), *ACTB* (β-actin; forward 5′-CTG GAA CGG TGA AGG TGA CA-3′, reverse 5′-AAG GGA CTT CCT GTA ACA ATG CA-3′), and *GAPDH* (glyceraldehyde-3-phosphate dehydrogenase; forward 5′-AGG TCG GTG TGA ACG GAT TTG-3′, reverse 5′-TGT AGA CCA TGT AGT TGA GGT CA-3′). TaqMan probes for *REN* (prorenin) were purchased from Thermo Fisher Scientific. Real-time qPCR was performed using the GoTaq qPCR Master mix (Promega), THUNDERBIRD Probe qPCR Mix (TOYOBO), and StepOne plus Systems (Thermo Fisher Scientific).

### Statistical analyses

All the results are expressed as the mean ± SEM (standard error of the mean). Statistical analyses were performed using Student’s t test following the analysis of variance (ANOVA), Fisher’s exact test and Spearman rank correlation. Differences between means were considered statistically significant when *p* values were < 0.05.

## Results

### Patients’ clinical characteristics

A total of 40 subjects with type 2 diabetes complicated by PDR (n = 20, the PDR group) and without diabetes suffering from idiopathic retinopathies (n = 20, the non-DM group) were included in the analysis. The mean ± SEM age was 63.35 ± 0.96 years in the non-DM group and 62.40 ± 1.95 years in the PDR group, with no significant difference between groups (*p* = 0.665). The male to female ratio was 10:10 in the non-DM group, and 7:13 in the PDR group, with no significant difference (*p* = 0.523). Among basal characteristics of participating patients, there were no significant differences between the non-DM and PDR groups except for random blood sugar (RBS) levels (non-DM = 105.90 ± 2.95 mg/dl, PDR = 159.40 ± 12.02 mg/dl, *p* < 0.001), serum creatinine (sCr) levels (non-DM = 0.71 ± 0.03 mg/dl, PDR = 1.00 ± 0.11 mg/dl, *p* < 0.05) and Logarithm of the minimum angle of resolution (Log MAR) visual acuity (non-DM = 0.71 ± 0.09, PDR = 1.28 ± 0.16, *p* < 0.01) (Table 1). As compared to the non-DM controls, the PDR patients enrolled in this study were shown to be characterized by hyperglycemia, renal dysfunction, and vision loss.

### Upregulation of s(P)RR, prorenin and activated prorenin protein levels in the plasma of PDR patients

**Fig 1:**
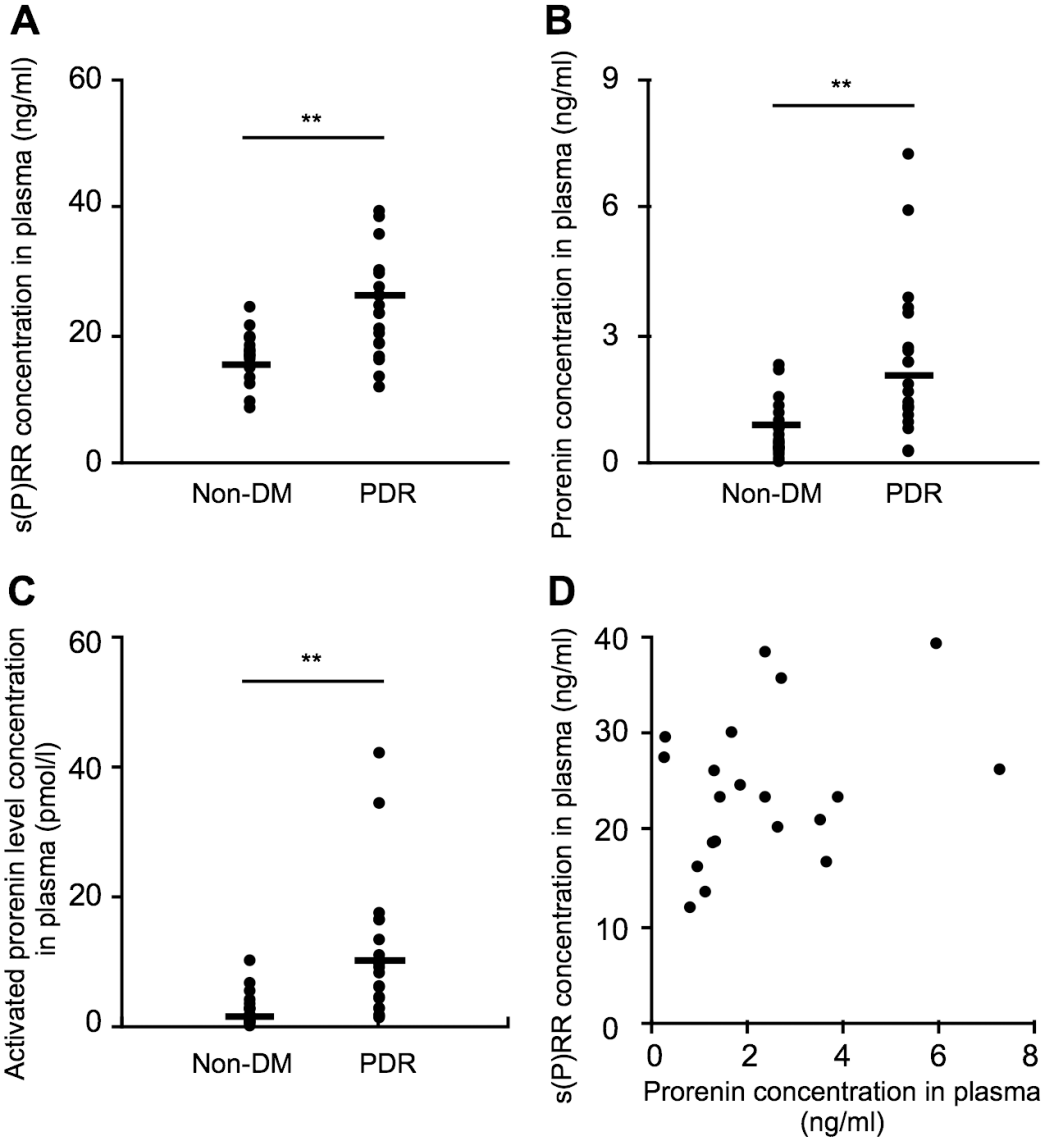
Upregulation of s(P)RR, prorenin and activated prorenin protein levels in the plasma of PDR patients. Protein levels of s(P)RR (A), prorenin (B), and activated prorenin (C) in the plasma of the non-DM and PDR subjects. Black symbols indicate individual samples. n = 20 in each group, **p < 0.01, Student’s t test. (D) Correlation between s(P)RR and prorenin was not detected in the plasma of patients with PDR (n = 20, Spearman rank correlation).

Elevated levels of plasma s(P)RR and prorenin have been reported to be associated with the pathogenesis of several diseases such as preeclampsia and renal dysfunction with heart failure [13–20]. To investigate the involvement of these RAS initiators in the blood circulatory system, we performed ELISA experiments using the plasma from PDR and non-DM subjects. As compared to the non-DM group [s(P)RR = 16.60 ± 0.85 ng/ml; prorenin = 0.77 ± 0.14 ng/ml], plasma protein levels of s(P)RR and prorenin significantly increased in the PDR group [s(P)RR = 24.34 ± 1.72 ng/ml, *p* < 0.01; prorenin = 2.33 ± 0.40 ng/ml, *p* < 0.01] (Fig 1A and 1B). Importantly, activated prorenin levels in the PDR samples (11.04 ± 2.40 pmol/l, *p* < 0.01) significantly increased compared with the non-DM controls (2.48 ± 0.57 pmol/l) (Fig 1C), indicative of s(P)RR-induced RAS activation in the systemic circulation of patients with PDR. Plasma protein levels of s(P)RR were not correlated with those of prorenin in the PDR group (*p* = 0.17, r = 0.32; Fig 1D), suggesting a potential difference between s(P)RR and prorenin in their upstream regulatory stimuli.

### Correlations between plasma s(P)RR and renal dysfunction parameters

**Fig 2:**
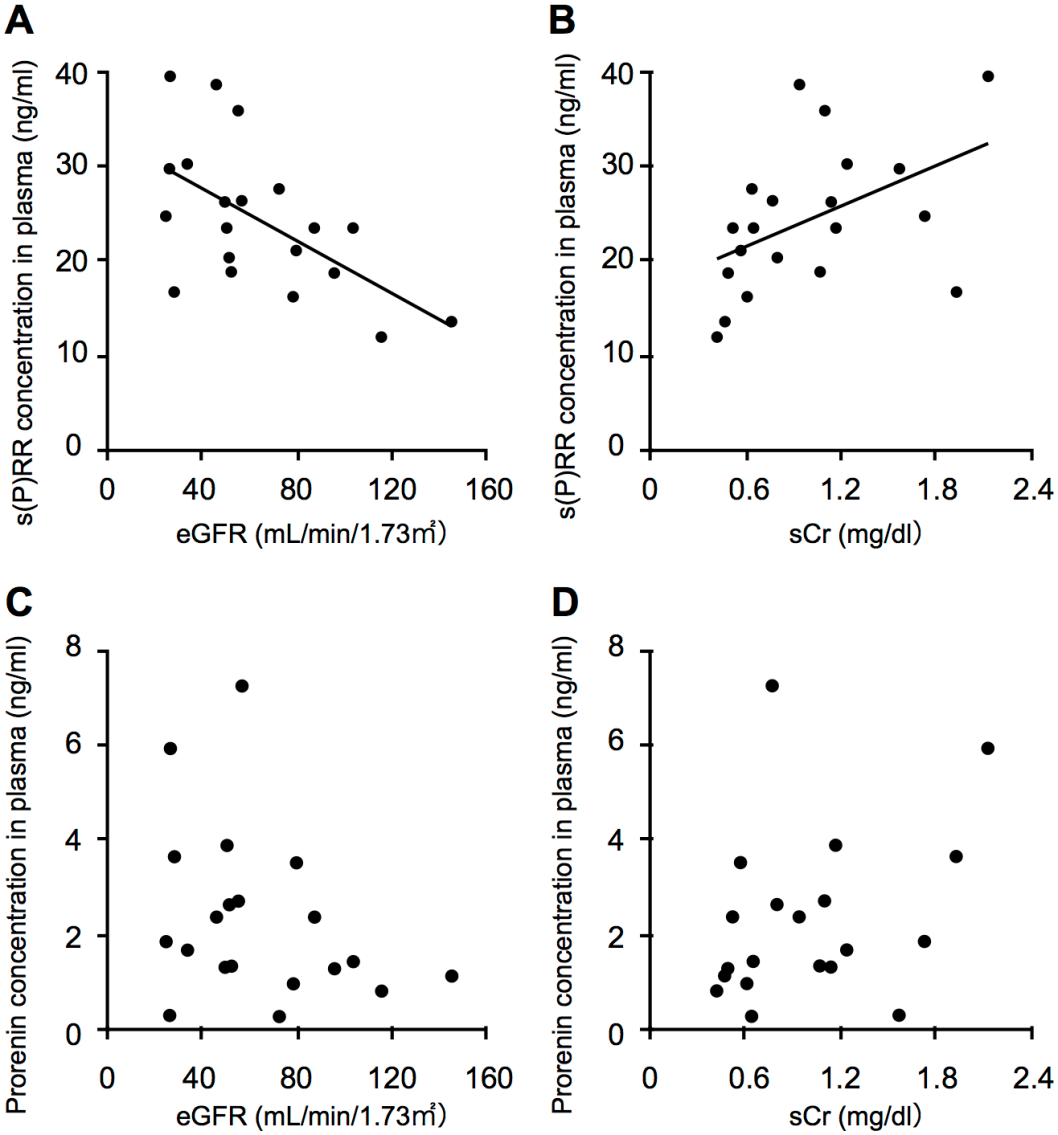
Correlations between plasma s(P)RR and renal dysfunction parameters. Correlations between s(P)RR and renal dysfunction parameters eGFR (A) and sCr (B) in the plasma of patients with PDR. (n = 20, Spearman rank correlation). Correlations between prorenin and renal dysfunction parameters eGFR (C) and sCr (D) were not detected in the plasma of patients with PDR (n = 20, Spearman rank correlation).

Previous studies showed that increased plasma s(P)RR levels were negatively correlated with estimated glomerular filtration rate (eGFR) in patients with heart and kidney failure and essential hypertension [13–15]. In the PDR group, plasma s(P)RR levels showed significant negative and positive correlations with eGFR (*p* = 0.006, r = -0.59) and sCr (*p* = 0.037, r = 0.47), respectively (Fig 2A and 2B), out of clinical parameters examined (Table 1). In contrast, there were no correlations between these renal dysfunction parameters and the plasma levels of prorenin (eGFR, *p* = 0.160, r = -0.326; sCr, *p* = 0.164, r = 0.324) (Fig 2C and 2D) or activated prorenin (eGFR, *p* = 0.088, r = -0.39; sCr, *p* = 0.066, r = 0.42) (data not shown), suggesting a susceptibility of s(P)RR over prorenin and activated prorenin to the kidney changes in diabetes.

### Inflammatory and angiogenic molecules in plasma of PDR patients

**Fig 3:**
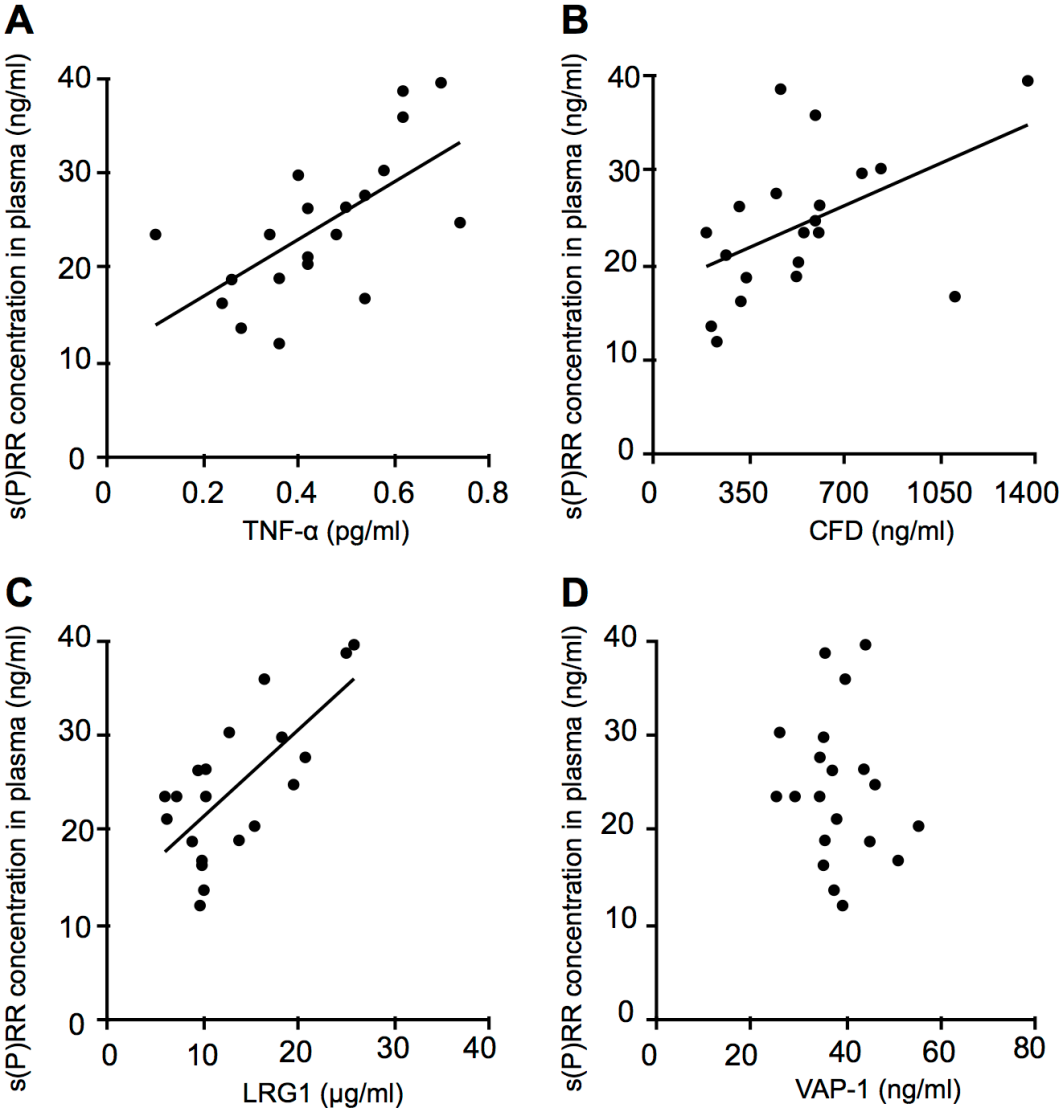
Correlations between s(P)RR and inflammatory mediators elevated in the plasma of PDR patients. Correlations between s(P)RR and inflammatory mediators TNF-α (A), CFD (B), LRG1 (C) and VAP-1 (D) in the plasma of patients with PDR (n = 20, Spearman rank correlation).

**Fig 4:**
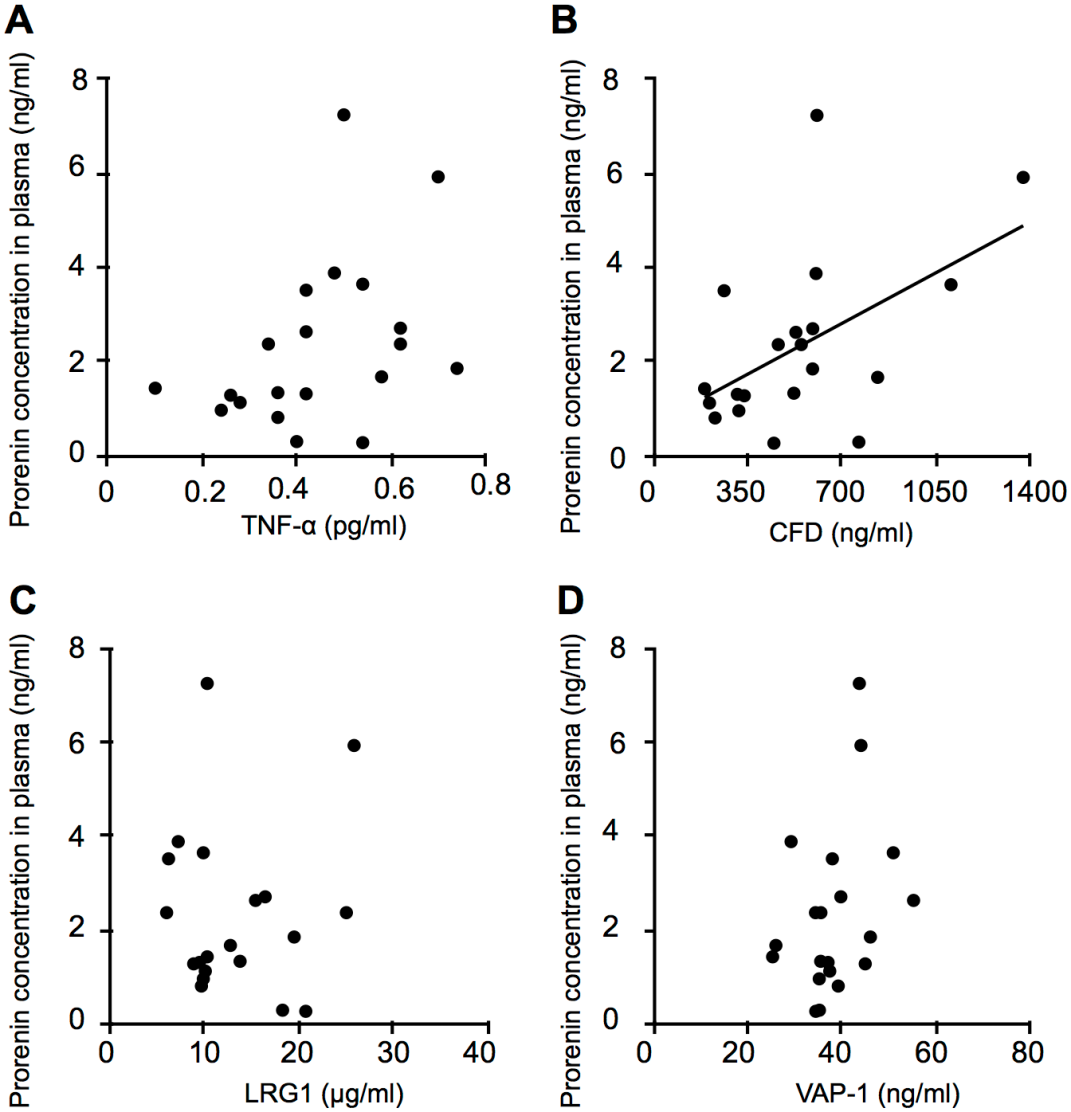
Correlations between prorenin and inflammatory mediators elevated in the plasma of PDR patients. Correlations between prorenin and inflammatory mediators TNF-α (A), CFD (B), LRG1 (C) and VAP-1 (D) in the plasma of patients with PDR (n = 20, Spearman rank correlation).

**Fig 5:**
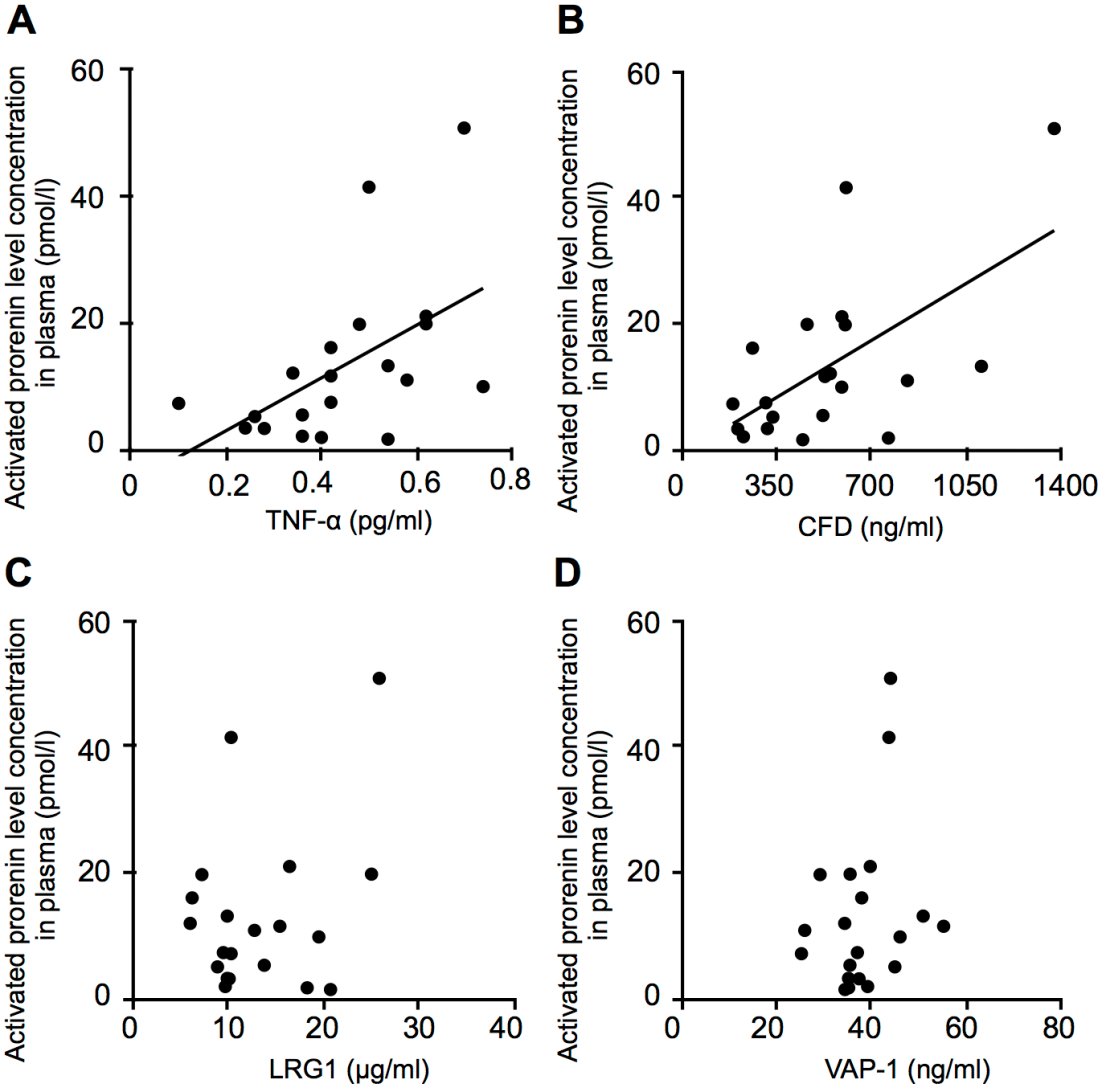
Correlations between activated prorenin and inflammatory mediators elevated in the plasma of PDR patients. Correlation between activated prorenin and inflammatory mediators TNF-α (A), CFD (B), LRG1 (C) and VAP-1 (D) in the plasma of patients with PDR (n = 20, Spearman rank correlation).

During long-standing hyperglycemia in diabetes, excessive glucose leads to the activation of several pathways such as the polyol pathway, protein kinase C activation, and the superoxide pathway [23]. Activation of these pathways leads to chronic inflammation in target organs with upregulation of inflammation-related molecules such as TNF-α and VAP-1, both of which proved to be elevated in the plasma of patients with diabetes [23–27]. To investigate systemic factors related to s(P)RR increased in the plasma of PDR patients, we measured protein levels of various molecules responsible for inflammation and neovascularization. Plasma levels of TNF-α, CFD, LRG1, and VAP-1 were significantly higher in the PDR group than in the non-DM group (TNF-α = 0.45 ± 0.04 vs. 0.34 ± 0.02 pg/ml, *p* = 0.015; CFD = 542.81 ± 67.37 vs. 362.40 ± 22.36 ng/ml, *p* = 0.018; LRG1 = 13.28 ± 1.33 vs. 7.68 ± 0.93 μg/ml, *p* = 0.001; VAP-1 = 38.41 ± 1.69 vs. 22.25 ± 1.04 ng/ml, *p* < 0.001), out of the inflammatory and angiogenic factors examined (Table 2). CFD regulates a key step in the activation of the alternative complement pathway and complement-mediated chronic inflammation, which is known to be associated with diabetic microvascular complications [28–30]. LRG1 is known to be a biomarker for various inflammatory diseases, including rheumatoid arthritis, ulcerative colitis, and asthma [31–33], and also functions as an endothelial cell mitogen for tumor and retinal neovascularization [34, 35]. The results indicated the potential correlations of these inflammatory mediators with the RAS initiators s(P)RR, prorenin and activated prorenin, which were evaluated in the following analyses (Figs 3–5).

**Table 2:** Plasma concentrations of inflammatory and angiogenic molecules.

	Non-DM	PDR	*p* value
**TNF-α (pg/ml)**	0.34 ± 0.02	0.45 ± 0.04	0.015
**CFD (ng/ml)**	362.40 ± 22.36	542.81 ± 67.37	0.018
**LRG1 (μg/ml)**	7.68 ± 0.93	13.28 ± 1.33	0.001
**VAP-1 (ng/ml)**	22.25 ± 1.04	38.41 ± 1.69	< 0.001
**MCP-1/CCL2 (pg/ml)**	15.27 ± 2.40	15.07 ± 1.32	0.945
**IFN-γ (pg/ml)**	1.26 ± 0.01	1.28 ± 0.01	0.097
**IL-6 (pg/ml)**	0.22 ± 0.03	0.25 ± 0.04	0.455
**VCAM-1 (ng/ml)**	144.87 ± 24.28	376.79 ± 117.57	0.067
**ICAM-1 (ng/ml)**	3.00 ± 0.96	8.29 ± 4.15	0.229
**FGF-1 (pg/ml)**	1.99 ± 0.38	1.62 ± 0.12	0.370
**FGF-2 (pg/ml)**	1.20 ± 0.06	1.13 ± 0.05	0.363
**PlGF (pg/ml)**	0.04 ± 0.02	0.06 ± 0.02	0.419
**Endostatin (ng/ml)**	3.63 ± 0.32	4.31 ± 0.54	0.286
**VEGF (pg/ml)**	0.41 ± 0.02	0.44 ± 0.04	0.605

TNF, tumor necrosis factor; CFD, complement factor D; LRG1, leucine-rich α-2-glycoprotein 1; VAP, vascular adhesion protein; MCP, monocyte chemoattractant protein; CCL, C-C motif ligand; IFN, interferon; IL, interleukin; VCAM, vascular cell adhesion molecule; ICAM, intercellular adhesion molecule; FGF, fibroblast growth factor; PlGF, placental growth factor; VEGF, vascular endothelial growth factor. Results are expressed as mean ± SEM. n = 20 in each group. Statistical analyses were performed using Student’s t test.

### Correlations between RAS initiators and inflammatory mediators elevated in the plasma of PDR patients

Correlations between s(P)RR and the inflammation-related molecules TNF-α, CFD, LRG1 and VAP-1, all of which increased in the plasma of PDR patients (Table 2), were assessed. Plasma protein levels of TNF-α, CFD and LRG1 were significantly correlated with those of s(P)RR in PDR patients (TNF-α, *p* = 0.003, r = 0.64; CFD, *p =* 0.027, r = 0.49; LRG1, *p* = 0.0005, r = 0.71) (Fig 3A–3C). However, no correlation between plasma s(P)RR and VAP-1 was found in PDR (*p* = 0.532, r = -0.15) (Fig 3D).

Next, we examined correlations between prorenin and these 4 molecules. Plasma CFD levels showed a significant correlation with prorenin levels in PDR patients (*p =* 0.020, r = 0.517), but not others (TNF-α, *p* = 0.07, r = 0.410; LRG1, *p* = 0.888, r = 0.034; VAP-1, *p =* 0.152, r = 0.333) (Fig 4A–4D).

Finally, correlations between activated prorenin and these 4 molecules were evaluated. Plasma protein levels of TNF-α and CFD, but not LRG1 or VAP-1, were significantly correlated with activated prorenin levels in PDR patients (TNF-α, *p =* 0.017, r = 0.527; CFD, *p* = 0.005, r = 0.603; LRG1, *p* = 0.185, r = 0.309; VAP-1, *p =* 0.311, r = 0.239) (Fig 5A–5D). These results (Figs 3–5) showed that VAP-1 was the only protein having no correlation with any of these RAS initiators, suggesting its RAS-independent regulatory mechanism.

To examine mRNA expression of the RAS-related inflammatory molecules (TNF-α, CFD and LRG1), (P)RR (gene code: *ATP6AP2*) and prorenin (gene code: *REN*) in peripheral whole blood samples, we carried out real-time qPCR analyses. Real-time qPCR analyses showed that *TNFA* and *CFD* mRNA levels were significantly higher in the PDR group (*CFD*, fold change = 1.65, *p* < 0.05; *TNFA*, fold change = 1.91, *p* < 0.01) than in the non-DM group (S1A and S1B Fig). There were no significant differences in *LRG1* or *ATP6AP2* gene expression between the groups (*LRG1*, fold change = 1.37, *p* > 0.05; *ATP6AP2*, fold change = 1.07, *p* > 0.05) (S1C and S1D Fig). *REN* mRNA levels were not detectable. The results suggested that diabetes-induced upregulation of s(P)RR, prorenin and LRG-1 did not stem from circulating leukocytes.

### TNF-α- and glucose-induced expression of RAS initiators in retinal vascular endothelial cells

**Fig 6:**
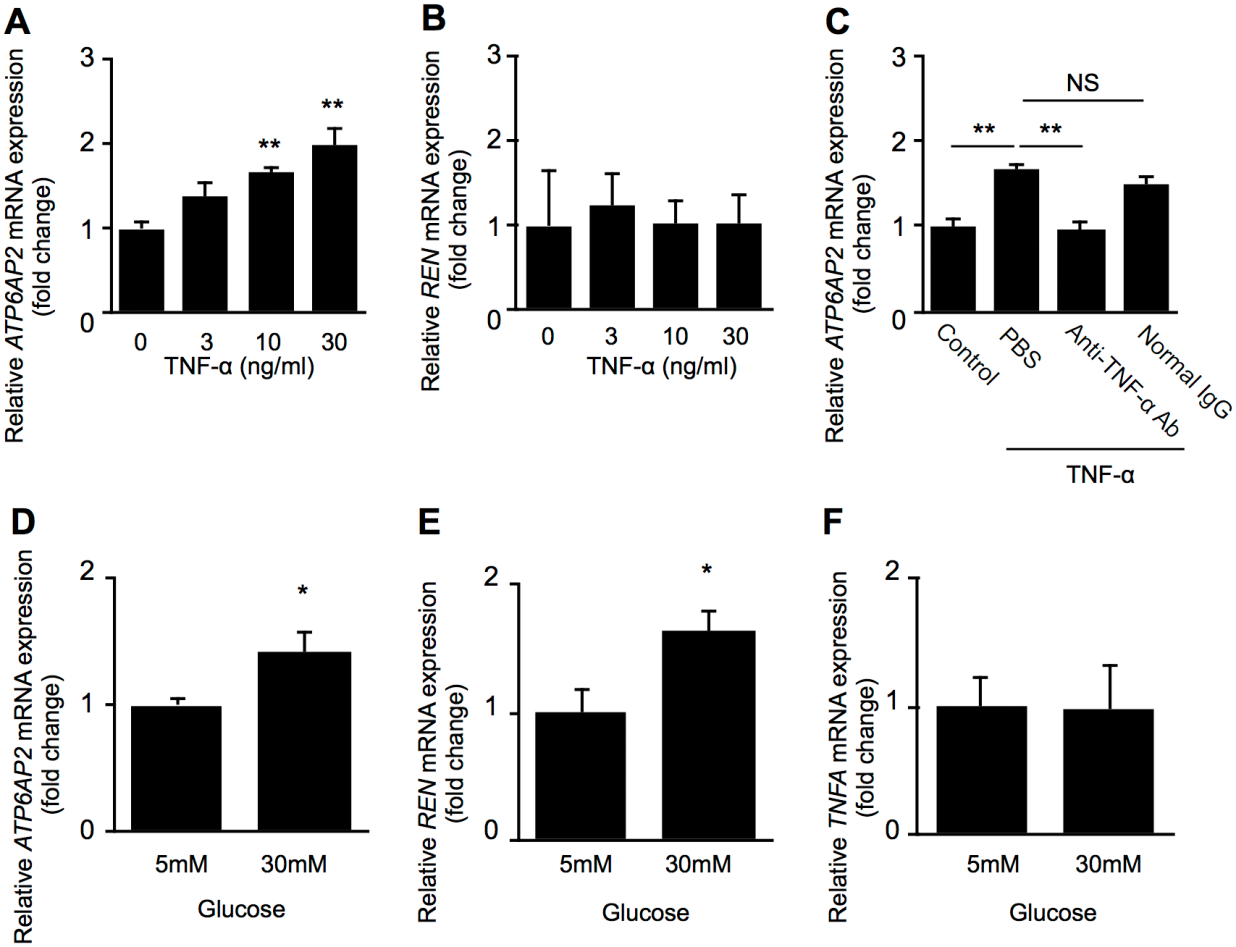
TNF-α- and glucose-induced expression of RAS initiators in retinal vascular endothelial cells. (A, B) *ATP6AP2* and *REN* mRNA expression levels in HRMECs stimulated by inflammatory mediators. After serum starvation, HRMECs were treated with recombinant human TNF-α (0, 3, 10 and 30 ng/ml) for 24 hours and processed for analysis to detect mRNA expression levels of *ATP6AP2* (A) and *REN* (B). n = 6, **p < 0.01, Student’s t test. (C) For TNF-α neutralization bioassay, recombinant human TNF-α at 10 ng/ml was pre-incubated with 200 ng/ml of rabbit anti-TNF-α neutralizing antibody. n = 6, **p < 0.01, Student’s t test. (D-F) HRMECs were incubated with the medium containing 30-mM glucose for 72 h, and *ATP6AP2* (D), *REN* (E) and *TNFA* (F) gene expression levels were analyzed. n = 6, *p < 0.05, Student’s t test.

Previously, our surgical sample data on human PDR eyes demonstrated tissue co-localization of both (P)RR and prorenin in neovascular endothelial cells harboring s(P)RR-shedding proteases such as furin and ADAM19, suggesting that the major source of s(P)RR elevated in PDR eyes was attributable, at least in part, to retinal vascular endothelial cells [4]. In agreement with the present data showing that diabetes-associated plasma s(P)RR elevation did not depend on leukocytes (S1D Fig), we used retinal vascular endothelial cells in the following experiments (Fig 6, S2 and S3 Figs), in order to detect diabetes-related upstream stimuli for the RAS initiators (P)RR and prorenin.

To study cause-effect relationships between the RAS initiators and the RAS-related inflammatory molecules (TNF-α, CFD and LRG1), we examined (P)RR/*ATP6AP2* and prorenin/*REN* mRNA expression levels treated with these molecules in HRMECs. Although neither administration with CFD nor LRG1 to HRMECs affected *ATP6AP2* or *REN* mRNA levels (S2A–S2D Fig), we found that TNF-α stimulation to HRMECs significantly increased the mRNA levels of *ATP6AP2* (3 ng/ml, fold change = 1.36, *p* > 0.05; 10 ng/ml, fold change = 1.66, *p* < 0.01; 30 ng/ml, fold change = 1.99, *p* < 0.01), but not *REN* (3 ng/ml, fold change = 1.23, *p* > 0.05; 10 ng/ml, fold change = 1.03, *p* > 0.05; 30 ng/ml, fold change = 1.02, *p* > 0.05), compared to those of controls in a dose-dependent manner (Fig 6A and 6B), in consistence with the absent correlation between s(P)RR and prorenin in the plasma of PDR patient (Fig 1D).

To confirm TNF-α-induced *ATP6AP2* mRNA expression in HRMECs, we used anti-TNF-α neutralizing antibody to examine the expression levels of *ATP6AP2*. TNF-α-induced upregulation of *ATP6AP2* gene expression was significantly suppressed by pretreatment with anti-TNF-α neutralizing antibody (fold change = 0.96, *p* < 0.01) compared to control normal IgG treatment (fold change = 1.51) (Fig 6C). In addition, we investigated whether administration of prorenin or angiotensin II with or without their receptor blockers to HRMECs changes *TNFA*, *CFD* and *LRG1* mRNA levels. However, there were no significant differences in gene expression under any of the conditions (*p* > 0.05) (S3A–S3C Fig).

It has been reported that (P)RR and prorenin were upregulated in podocytes and mesangial cells treated with high glucose [36, 37]. We examined whether *ATP6AP2* and *REN* mRNA levels change in HRMECs under high-glucose stimulation. Consequently, both *ATP6AP2* and *REN* mRNA expression levels were higher in HRMECs applied with high glucose at 30 mM than in those with osmolality-controlled 5-mM glucose (*ATP6AP2*, fold change = 1.41, *p* < 0.05; *REN*, fold change = 1.65, *p* < 0.05) (Fig 6D and 6E), but no change was detected in *TNFA* gene expression (Fig 6F).

## Discussion

**Fig 7:**
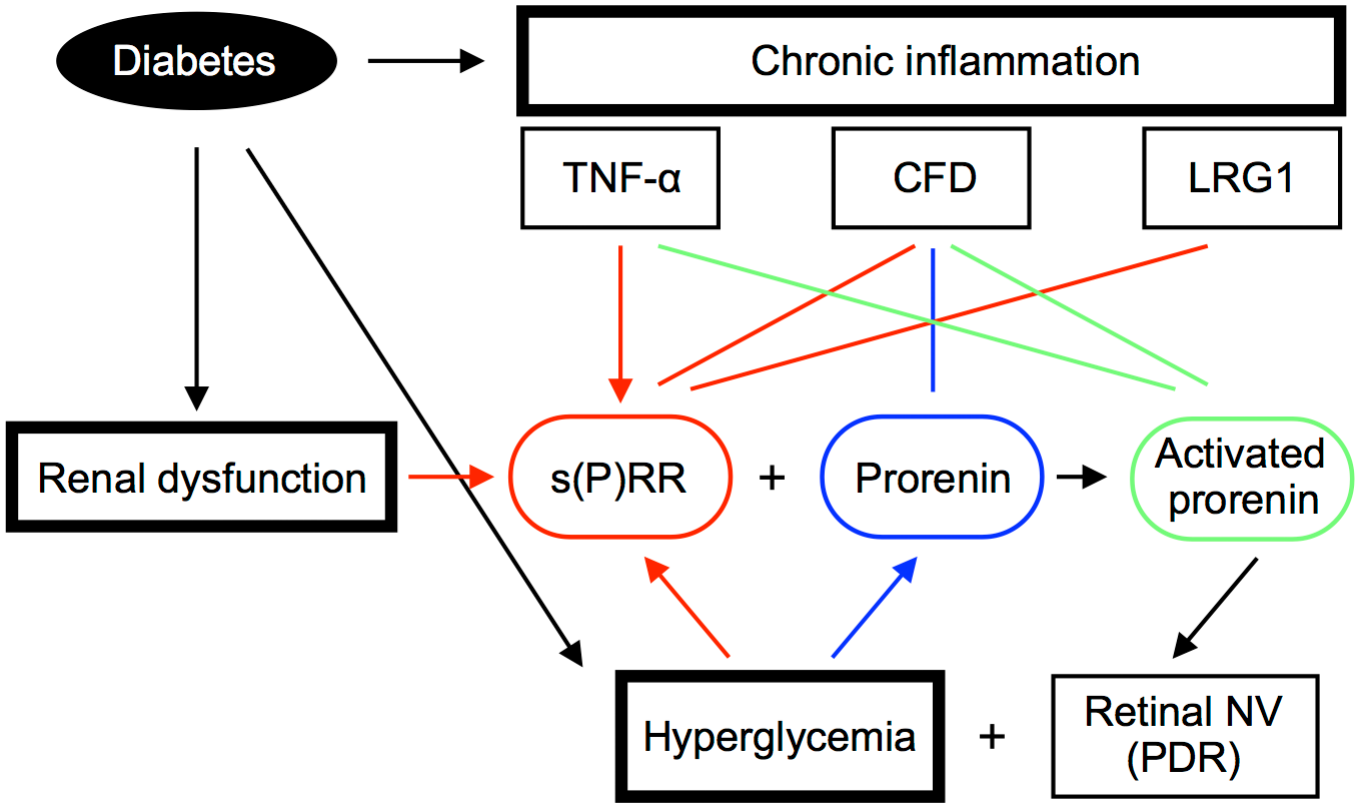
Association of plasma s(P)RR with chronic inflammation, renal dysfunction, and hyperglycemia in patients with PDR. A schema showing diabetes-induced factors such as chronic inflammation, renal dysfunction, and hyperglycemia as the potential regulators of plasma s(P)RR and prorenin levels, thus initiating the RAS activation to enhance retinal neovascularization (NV), the hallmark of PDR. Retinal NV, in turn, functions as a cellular source of these RAS initiators under hyperglycemia, generating the vicious cycle of RAS and PDR. Arrows indicate cause-effect relations, and lines represent correlations.

This study demonstrates, for the first time to our knowledge, the following important data on several systemic factors related to s(P)RR in the blood circulation of patients with PDR. Plasma levels of the RAS initiators s(P)RR, prorenin and activated prorenin increased in the PDR group (Fig 1), which was characterized by hyperglycemia, renal dysfunction, and vision loss (Table 1), when compared with the non-DM group. Renal dysfunction parameters were correlated with plasma s(P)RR, but not prorenin or activated prorenin (Fig 2). Of various biochemical mediators examined, TNF-α, CFD, LRG1 and VAP-1 increased in the plasma of PDR patients (Table 2). Importantly, some of these inflammation-related molecules were correlated with the RAS initiators: s(P)RR with TNF-α, CFD and LRG1 (Fig 3); prorenin with CFD (Fig 4); activated prorenin with TNF-α and CFD (Fig 5). Among these RAS-related inflammatory mediators, TNF-α stimulation to retinal vascular endothelial cells upregulated the mRNA expression of (P)RR but not prorenin, while stimulation with high glucose enhanced both (P)RR and prorenin expression (Fig 6). These findings suggested close relationships between the RAS initiators, especially s(P)RR, and diabetes-induced factors including chronic inflammation, renal dysfunction, and hyperglycemia (Fig 7).

The pathogenesis of diabetes includes chronic inflammation due to hyperglycemia-induced activation of monocytes, which produce various growth factors and cytokines [26, 27]. TNF-α is one of the major cytokines responsible for chronic inflammation in diabetes, and plays a pivotal role in the development of renal and retinal microvascular complications [23, 26–28]. Importantly, systemic (*i*.*e*., intravenous) injections of the anti-TNF-α antibody infliximab to patients with diabetic macular edema led to significant visual function gains [38, 39]; however, little or no efficacious changes were achieved by local (*i*.*e*., intravitreal) application with either of the two different TNF-α inhibitors infliximab or adalimumab [40]. These results suggested that diabetes-induced TNF-α elevated in the systemic circulation, rather than that locally in the eye, contributes to the pathogenesis of retinal microvascular complications. This is also supported by our interpretation of the potential mechanism (Fig 7) whereby systemic TNF-α causes the tissue RAS activation in the diabetic retina; therefore, blocking intraocular TNF-α only topically [40] was likely to be insufficient in suppressing the activity of retinopathy. Notably, the present study is the first to suggest TNF-α as an upstream regulatory factor for the induction of (P)RR, in accordance with the central role of TNF-α in diabetic chronic inflammation [23, 26–28].

Activation of the complement system is well known to engage in chronic inflammation in diabetes [28–30]. CFD, the rate-limiting protease triggering the alternative pathway, is also identical to adipsin, one of the adipokines, which improves the pancreatic β cell function, thus playing a protective role in diabetes [41]. Indeed, type 2 diabetic patients with β cell failure were reported to be less abundant in CFD than those without β cell failure [41]. The current results on the plasma CFD elevation in type 2 diabetic patients with PDR are consistent with previous data showing that type 2 diabetic patients with obesity had higher levels of CFD than healthy controls [42]. These findings suggested that systemic CFD levels fluctuate at different stages of diabetes according to its double-edged functions as a detrimental inflammatory mediator as well as a beneficial insulin regulator.

LRG1, a known biomarker for various inflammatory diseases [31–33], is also regarded as an angiogenic factor [34, 35]. The current investigation is the first to show the systemic elevation of LRG1 levels in diabetes. LRG1 was shown to localize exclusively in the vasculature of various human tissues including the eye and increase in the murine model of oxygen-induced ischemic retinopathy [35], which is characterized by retinal vaso-obliteration and neovascularizion, as always seen in PDR. Indeed, the intravitreal levels of LRG1 increased in PDR eyes [35], possibly due to hypoxia-induced local production, which may have affected the systemic elevation of LRG1 observed in our present study. It remains to be determined, however, whether diabetes per se (*i*.*e*., without retinopathy), like other inflammatory disorders [31–33], causes the upregulation of LRG1 in the systemic circulation.

Several studies have demonstrated that the prevalence of retinopathy is strongly associated with nephropathy in diabetes [43–45], linking low eGFR levels with the onset and progression of DR [46]. In patients with renal dysfunction, systemic s(P)RR levels were negatively correlated with eGFR [13, 14]. Ours is the first to report significant correlations between s(P)RR and renal dysfunction parameters eGFR and sCr levels in patients with diabetes. Importantly, (P)RR expression increased in the kidney of patients with diabetic nephropathy [47], and was shown to enhance renal production of inflammatory cytokines including TNF-α and IL-1β as a potential mechanism involved in the development of kidney disorder in the rodent model of streptozotocin-induced diabetes [48]. These results suggested that the elevated s(P)RR levels in the systemic circulation would not simply imply the maker of renal dysfunction, but also contribute to the pathogenesis of diabetic nephropathy. This may also be true with retinopathy, on the basis of a positive correlation between intravitreal s(P)RR levels and the angiogenic activity of PDR [4], though future studies are needed to investigate the influence of s(P)RR on other target organs.

Retinal neovessels in the fibrovascular tissue of PDR were shown to be immunopositive for (P)RR and prorenin [4], in consistence with the current results on the *in vitro* upregulation of (P)RR/*ATP6AP2* and prorenin/*REN* expression in retinal vascular endothelial cells stimulated with high glucose. Diabetes-associated hyperglycemia may work as an amplifier for systemic s(P)RR and prorenin levels by using retinal neovascular cells as the potential source of these RAS initiators, which in turn promote the angiogenic activity of PDR, so as to generate the vicious cycle of RAS and PDR (Fig 7). On the other hand, high glucose application has also proven to be the main inducer for (P)RR expression in kidney cell lines (*e*.*g*., mesangial cells and podocytes) [36, 37], in accordance with our current and previous data showing that s(P)RR protein levels were higher in the plasma than in the eye of patients with PDR [4]. Taken together with the following piles of evidence including the *in vitro* induction of (P)RR in glucose-stimulated renal cells [36, 37], the *in vivo* expression of (P)RR in the kidney of patients and animals with diabetic nephropathy [47, 48], the systemic elevation of s(P)RR in patients with kidney dysfunction and failure [13, 14, 16, 20], and the significant association of s(P)RR with renal dysfunction parameters (Fig 2); the currently observed increase in plasma s(P)RR levels in patients with PDR may have resulted mainly from renal rather than retinal production.

In conclusion, these findings suggested diabetes-induced factors such as chronic inflammation, renal dysfunction, and hyperglycemia as the potential regulators of systemic s(P)RR elevation, thus initiating the tissue RAS activation to enhance retinal neovascularization in PDR. The current study warrants future investigation into whether a combination of systemic interventions comprehensively targeting these diabetic parameters further improves visual prognosis in the long-term management of diabetic patients with retinopathy in addition to its local therapy.

## Supporting information

S1 Fig mRNA expression levels of inflammatory mediators and (P)RR in peripheral whole blood samples. Relative mRNA expression levels of *TNFA* (A), *CFD* (B), *LRG1* (C) and *ATP6AP2* (D) in peripheral whole blood from the non-DM and PDR subjects. n = 20 in each group, *p < 0.05, **p < 0.01, Student’s t test.
(PDF)

S2 Fig (P)RR*/ATP6AP2* and prorenin/*REN* mRNA in HRMECs treated with CFD or LRG1. (P)RR/*ATP6AP2* (A, C) and prorenin/*REN* (B, D) mRNA expression levels in HRMECs stimulated by inflammatory molecules. After serum starvation, HRMECs were treated with recombinant human CFD (A, B) or LRG1 (C, D) (0, 3, 10 and 30 ng/ml) for 24 h and processed for analysis to detect mRNA expression levels of *REN* and *ATP6AP2*. n = 6, Student’s t test.
(PDF)

S3 Fig *TNFA*, *LRG1* and *CFD* mRNA in HRMECs treated with prorenin or Ang II. Relative mRNA expression levels of *TNFA* (A), *CFD* (B), and *LRG1* (C) in HRMECs stimulated by prorenin or Ang II with or without those receptor antagonists. n = 6, Student’s t test. Ang II: angiotensin II.
(PDF)
