# Supplementary material for: Systemic factors related to soluble (pro)renin receptor in plasma of patients with proliferative diabetic retinopathy
Source: PLoS One. 2017 Dec 14;12(12):e0189696. doi: 10.1371/journal.pone.0189696 (PMC5730163; doi:10.1371/journal.pone.0189696)
Supplement: S1 Fig — Relative mRNA expression levels of TNFA (A), CFD (B), LRG1 (C) and ATP6AP2 (D) in peripheral whole blood from the non-DM and PDR subjects. n = 20 in each group, *p < 0.05, **p < 0.01, Student’s t test. (PDF) [file pone.0189696.s001.pdf]

Hase et al., S1 Fig

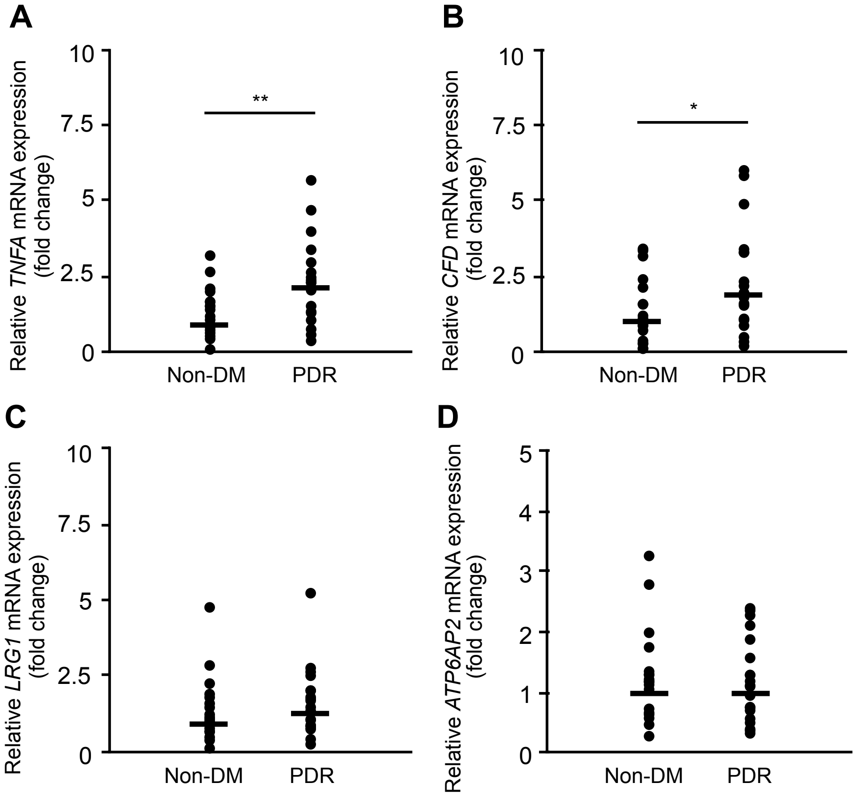

S1 Fig. mRNA expression levels of inflammatory mediator and RAS initiators in peripheral whole blood samples.

Relative RNA expression levels of *TNFA* (A), *CFD* (B), *LRG1* (C) and *ATP6AP2* (D) in peripheral whole blood from the non-DM and PDR subjects. n = 20 in each group, *$p < 0.05$, and **$p < 0.01$. Statistical analysis was performed using the Student's t test following the ANOVA.
