# Supplementary material for: Systemic factors related to soluble (pro)renin receptor in plasma of patients with proliferative diabetic retinopathy
Source: PLoS One. 2017 Dec 14;12(12):e0189696. doi: 10.1371/journal.pone.0189696 (PMC5730163; doi:10.1371/journal.pone.0189696)
Supplement: S2 Fig — (P)RR/ATP6AP2 (A, C) and prorenin/REN (B, D) mRNA expression levels in HRMECs stimulated by inflammatory molecules. After serum starvation, HRMECs were treated with recombinant human CFD (A, B) or LRG1 (C, D) (0, 3, 10 and 30 ng/ml) for 24 h and processed for analysis to detect mRNA expression levels of REN and ATP6AP2. n = 6, Student’s t test. (PDF) [file pone.0189696.s002.pdf]

Hase et al., S2 Fig

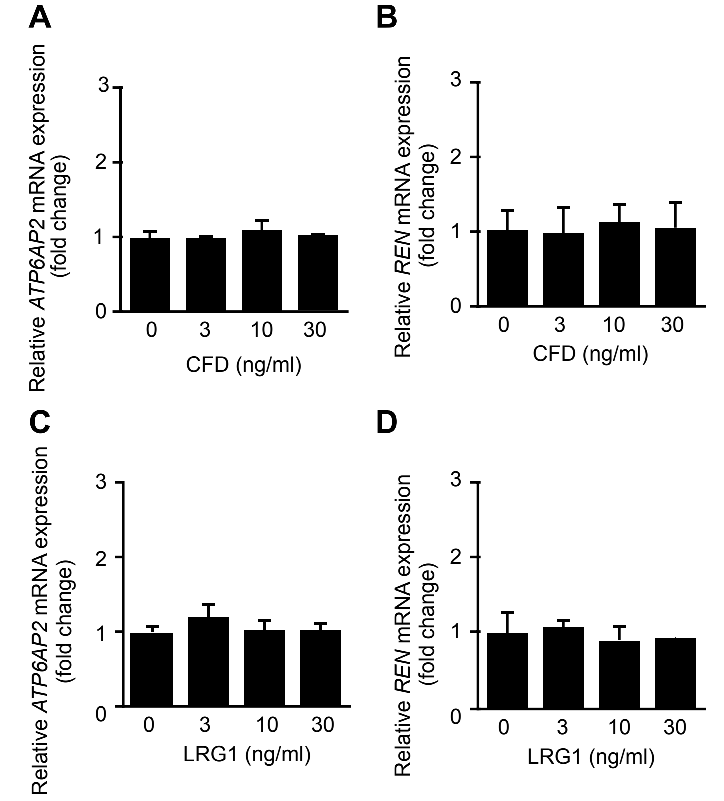

S2 Fig. (P)RR/*ATP6AP2* and Prorenin/*REN* mRNA in HRMECs treated with CFD or LRG1. (P)RR/*ATP6AP2* (A, C) and Prorenin/*REN* (B, D) mRNA expression levels in HRMECs stimulated by inflammatory molecules. After serum starvation, HRMECs were treated with recombinant human (A, B) CFD or (C, D) LRG1 (0, 3, 10 and 30 ng/ml) for 24 h and processed for analysis to detect mRNA expression levels of *REN* and *ATP6AP2*. n = 6. Statistical analysis was performed using the Student's t test following the ANOVA.
