# Supplementary material for: Systemic factors related to soluble (pro)renin receptor in plasma of patients with proliferative diabetic retinopathy
Source: PLoS One. 2017 Dec 14;12(12):e0189696. doi: 10.1371/journal.pone.0189696 (PMC5730163; doi:10.1371/journal.pone.0189696)
Supplement: S3 Fig — Relative mRNA expression levels of TNFA (A), CFD (B), and LRG1 (C) in HRMECs stimulated by prorenin or Ang II with or without those receptor antagonists. n = 6, Student’s t test. Ang II: angiotensin II. (PDF) [file pone.0189696.s003.pdf]

Hase et al., S3 Fig

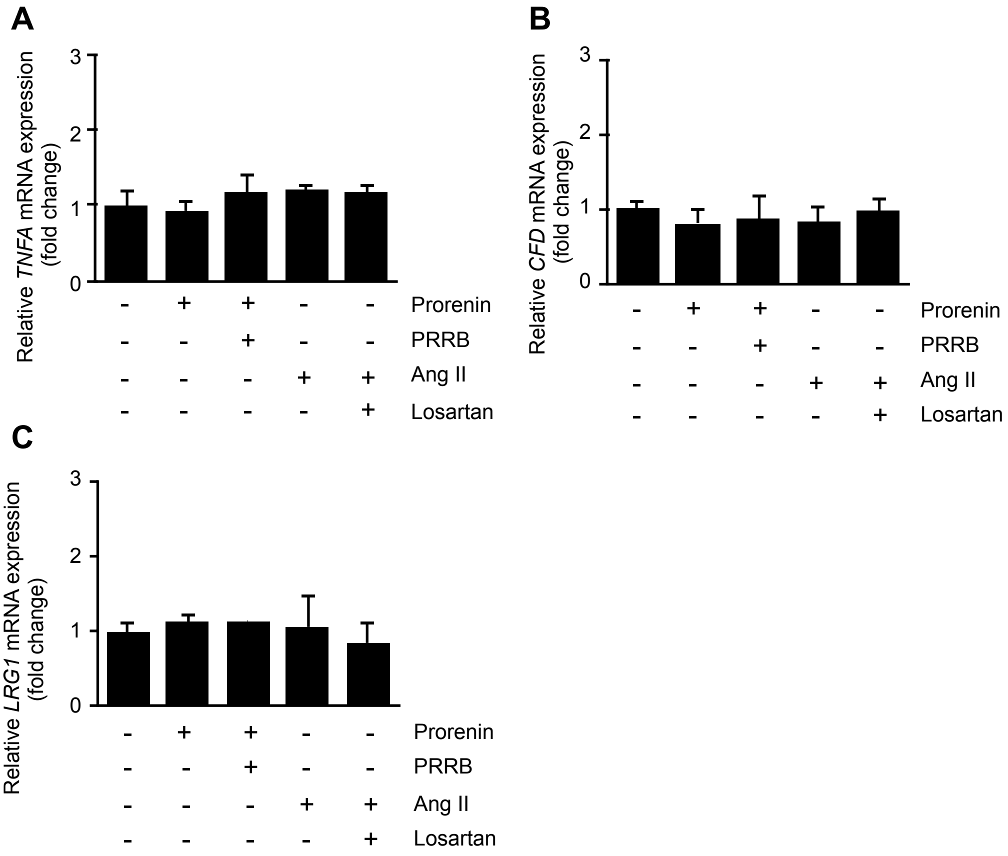

S3 Fig. *TNFA, LRG1* and *CFD* mRNA in HRMECs treated with prorenin and Ang II. Relative RNA expression levels of *TNFA* (A)*, CFD* (B)*, and LRG1* (C) in HRMECs stimulated by prorenin or Ang II with or without these receptor antagonists. n = 6. Ang II: angiotensin II. Statistical analysis was performed using the Student's t test following the ANOVA.
